# Glassy carbon, NIST Standard Reference Material (SRM 3600): hydrogen content, neutron vibrational density of states and heat capacity

**DOI:** 10.1107/S1600576718010828

**Published:** 2018-08-24

**Authors:** Ronald L. Cappelletti, Terrence J. Udovic, Hui Li, Rick L. Paul

**Affiliations:** a NIST Center for Neutron Research, National Institute of Standards and Technology, 100 Bureau Drive, MS 6102, Gaithersburg, MD 20899-6102, USA; b Department of Physics and Astronomy, Ohio University, Athens, OH 45701, USA; c Hall Radiation Center, 603 10th Street SE, Cedar Rapids, IA 52403, USA

**Keywords:** standard reference materials, glassy carbon, hydrogen content, neutron vibrational density of states, specific heat, graphite

## Abstract

Commercial glassy carbon plates being used as absolute intensity calibration standards in small-angle X-ray scattering applications (NIST SRM 3600) have been characterized in several recent publications. This contribution adds to the characterization by measuring the hydrogen content of a plate to be (4.8 ± 0.2) × 10^−4^ (mol H)/(mol C), and by measuring the vibrational spectrum by neutron inelastic scattering. The spectrum bears a strong resemblance to published measurements on graphite, allowing the identification of several spectral features. The measured spectrum is used to calculate the heat capacity of low-hydrogen-content glassy carbon for comparison with measurements reported here from 20 to 295 K.

## Abstract 2

Commercial glassy carbon plates (NIST SRM 3600) being used as absolute intensity calibration standards in small-angle X-ray scattering applications are further characterized in terms of hydrogen content, vibrational density of states and specific heat capacity.

## 1. Introduction   

A study (Zhang *et al.*, 2010 ▸) at the Advanced Photon Source (APS) of Argonne National Laboratory characterized glassy carbon plates obtained from Alfa Aesar^1^ for use in absolute intensity calibrations for small-angle X-ray scattering (SAXS) applications. Zhang *et al.* demonstrated that selected commercially available glassy carbon plates are sufficiently homogeneous and stable to serve as calibration standards and have made these plates available to several laboratories for this purpose. This material has been more formally developed as a new NIST Standard Reference Material (SRM 3600, 2017 ▸), as described in a recent article in this journal (Allen *et al.*, 2017 ▸). To further characterize these same plates, we have determined their hydrogen content and also performed neutron inelastic scattering measurements to determine the lattice vibrational spectrum. This, in turn, is used to calculate the heat capacity for comparison with measurements presented here on a similar sample.

## 2. Properties and structure of NIST SRM 3600   

The plates provided to us by APS for this study were obtained from Alfa Aesar (AA), Ward Hill, MA, USA, stock No. 30821. Batch details are given elsewhere (Allen *et al.*, 2017 ▸; SRM 3600, 2017 ▸; Zhang *et al.*, 2010 ▸). AA purchases its glassy carbon plates from Hochtemperatur-Werkstoffe GmbH (HTW), Thierhaupten, Germany, which designates them as SIGRADUR G (called Type-2 by AA) and provides tables of their properties (HTW, 2017 ▸). Highly impermeable, hard Type-2 materials have zero open porosity, a service temperature of 3273 K and a density of 1.42 g cm^−3^. Here we describe selected results from the literature appropriate to glassy carbon prepared similarly to AA Type-2. An early proposal based on X-ray diffraction measurements (Franklin, 1951 ▸) was that glassy carbon produced by pyrolysis at 3273 K be classified as non-graphitizing carbon. Subsequent neutron elastic scattering measurements (Mildner, 1974 ▸) out to a momentum transfer *Q* = 25 Å^−1^ were used to produce a radial distribution function to provide evidence that glassy carbon treated at 3273 K contains very few tetrahedral bonds. In a later analysis (Mildner & Carpenter, 1982 ▸), peaks were reported at 1.425, 2.35 and 2.83 Å, and a shoulder at 3.415 Å. The three peaks correspond to the C1—C2, C1—C3 and C1—C4 distances on a C_6_ hexagon. The shoulder is interpreted as the interlayer C—C distance seen in graphite. More recently, a study (Jurkiewicz *et al.*, 2017 ▸) was performed on a series of glassy carbons produced from a furfuryl alcohol precursor heat-treated to temperatures ranging from 1073 to 2773 K. The material produced at 2773 K, GC2500, may be expected to most closely resemble the higher-temperature AA Type-2 material and the materials studied by Mildner & Carpenter. X-ray and neutron diffraction measurements were used by Jurkiewicz *et al.* to provide structure factors and pair distribution functions, which they found to be in substantial agreement with each other. These were then compared with model calculations based on turbostratically amassed, curved graphene fragments containing specific defects and relaxed *via* molecular dynamics methods to produce a variety of structures, including parts resembling fullerenes. In brief, the models of Jurkiewicz *et al.* appear to provide a plausible explanation for the hardness of glassy carbons, their occluded porosity, their chemical inertness and the approach to graphite-like fragments with increasing heat-treatment temperature.

### 2.1. Hydrogen content of NIST SRM 3600   

Since the cross section for scattering neutrons from hydrogen is more than an order of magnitude greater than that from carbon, hydrogen has a significant impact on SANS measurements; hence, knowledge of the hydrogen content is important in assessing the usefulness of these plates for intensity standards applications. Therefore, we measured the hydrogen content of one of the plates from the APS collection by thermal neutron prompt gamma activation analysis using the NGD spectrometer at the NIST Center for Neutron Research (NCNR) (Paul *et al.*, 2015 ▸). The plate was found to have a hydrogen content of 40 ± 2 mg kg^−1^, or (4.8 ± 0.2) × 10^−4^ (mol H)/(mol C), with a 95% confidence interval. This measurement is expected to be statistically representative of the plates supplied to us. For comparison, using a different technique, Albers *et al.* (2016 ▸) found 60 ± 20 mg kg^−1^ for a Type-2 glassy carbon (GC) plate purchased from AA. The low hydrogen content also gives us confidence that the vibrational spectrum to be discussed below exhibits no observable carbon–hydrogen vibrational features, and this is confirmed by comparison with measurements on graphite, also discussed below.

In addition, we have determined the hydrogen content of Type-1 beads purchased from AA (product No. 41497, lot Y198034, 630–1000 µm diameter). Type-1 material has a maximum service temperature of 1273 K. Using the same method, we found it to contain 3082 ± 150 mg kg^−1^, or (3.68 ± 0.18) × 10^−2^ (mol H)/(mol C), of hydrogen, which is approximately 77 times the hydrogen content of AA Type-2 material. This relatively large value is evidence that not all of the hydrogen was removed from the starting material at the lower pyrolysis temperature. We confirmed that the neutron inelastic scattering vibrational spectrum (not shown here) of these AA Type-1 beads shows the same characteristic carbon–hydrogen modes seen elsewhere (Parker *et al.*, 2013 ▸) and not seen in the AA Type-2 material described in this work.

## 3. Neutron inelastic scattering measurements on glassy carbon samples   

### 3.1. Methods and results of neutron scattering measurements   

Two sets of neutron inelastic scattering measurements were made on different samples of low-hydrogen glassy carbon. The first (Kamitakahara *et al.*, 1992 ▸; Kamitakahara, 1996 ▸) was made on a sample provided by Professor Jeffrey S. Lannin of Penn State University. It was manufactured from an unspecified high-temperature decomposition of an organic polymer by Sloan Inc. for use in an electron beam evaporation boat. This sample displayed an X-ray diffraction pattern resembling graphite, with very broad peaks consistent with crystalline order over ∼3 nm (Li & Lannin, 1992 ▸). Although neither the heat-treatment temperature of this material nor its hydrogen content is known by us, we expect that since it was part of an electron beam evaporation boat it was probably made of high-temperature material similar to AA Type-2 material, and, indeed, apart from a small band around 150 meV of low statistical significance, no other hydrogen vibrational features were reported by Kamitakahara. Neutron inelastic scattering measurements on this sample were made using the IN4 time-of-flight spectrometer at the Institut Laue–Langevin. The IN4 measurements, made at room temperature, were corrected for mulitphonon scattering. These measurements are expected to give a reasonable representation of the spectrum in the region below 50 meV because of adequate sampling over the momentum-transfer variable *Q*.

The second set of measurements, made at the NCNR and reported in this work, was taken on the AA Type-2 plates provided to us. The neutron vibrational density of states (VDOS) spectrum was measured using the Filter Analyzer Spectrometer (BT4-FANS) (Udovic *et al.*, 2008 ▸). The plates were enclosed in a thin-walled aluminium can and cooled to 4.2 K using a top-loading displex refrigerator. A Cu(220) monochromator with a 60′ pre- and 40′ post-collimation was used to provide an energy range from ∼30 to ∼250 meV with an energy resolution (full width at half-maximum) ranging smoothly from ∼2 meV at 30 meV to ∼16 meV at 200 meV.

**Figure 1:**
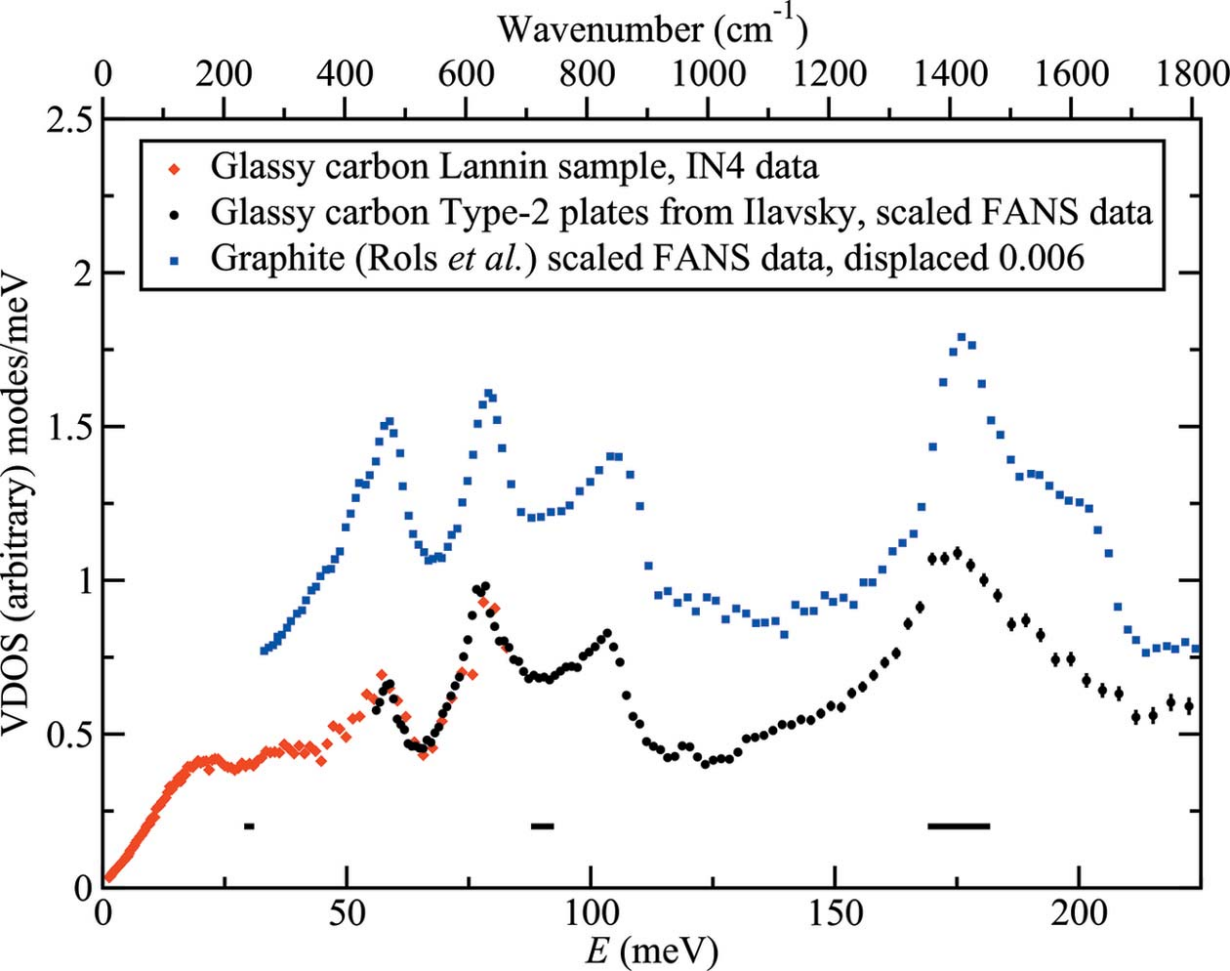
Composite neutron VDOS of low-hydrogen-content glassy carbon. The IN4 data on the Lannin sample (red diamonds) are from Kamitakahara *et al.* (1992 ▸). The BT4-FANS data (solid circles) on the APS plate samples were measured in this work and are scaled to match the IN4 data in the region between 60 and 90 meV. Error bars on the FANS data are ± one standard deviation of the mean. Horizontal bars represent instrumental energy resolution. Graphite measured by others (Rols *et al.*, 2000 ▸) on BT4-FANS (blue squares) is shown for comparison.

In order to compare the FANS measurements with the earlier IN4 measurements on the Lannin sample, the FANS data were scaled to match the peaks visible in both sets of measurements in the region from 60 to 90 meV. Fig. 1 ▸ shows the result. The observed spectrum is in substantial agreement with recent measurements (Albers *et al.*, 2016 ▸) that were made on an AA Type-2 glassy carbon plate.

### 3.2. Neutron scattering measurements on glassy carbon samples: discussion   

The glassy carbon neutron VDOS of these plates of Type-2 material from AA is in substantial agreement with earlier measurements on the Lannin sample reported by Kamitakahara, differing in some details, mainly in the weighting of the three main peaks below 125 meV. On the other hand, the plates display a strong resemblance to powdered graphite. A comparison is made with the measurements of Rols *et al.* (2000 ▸) in Fig. 1 ▸. (The region below 50 meV in the graphite data is not expected to represent the neutron VDOS very well because of the limited region of the *Q* variable sampled in the filter-analyzer method used at BT4.)

As seen in Fig. 1 ▸, the major features of GC, neglecting the weighting, line up remarkably well with graphite. It is by now well known that forms of carbon based on the fullerenes share many graphite-like features, consistent with graphitic structural motifs in these materials, and the same can be said of GC, as noted by Jurkiewicz *et al.*

**Figure 2:**
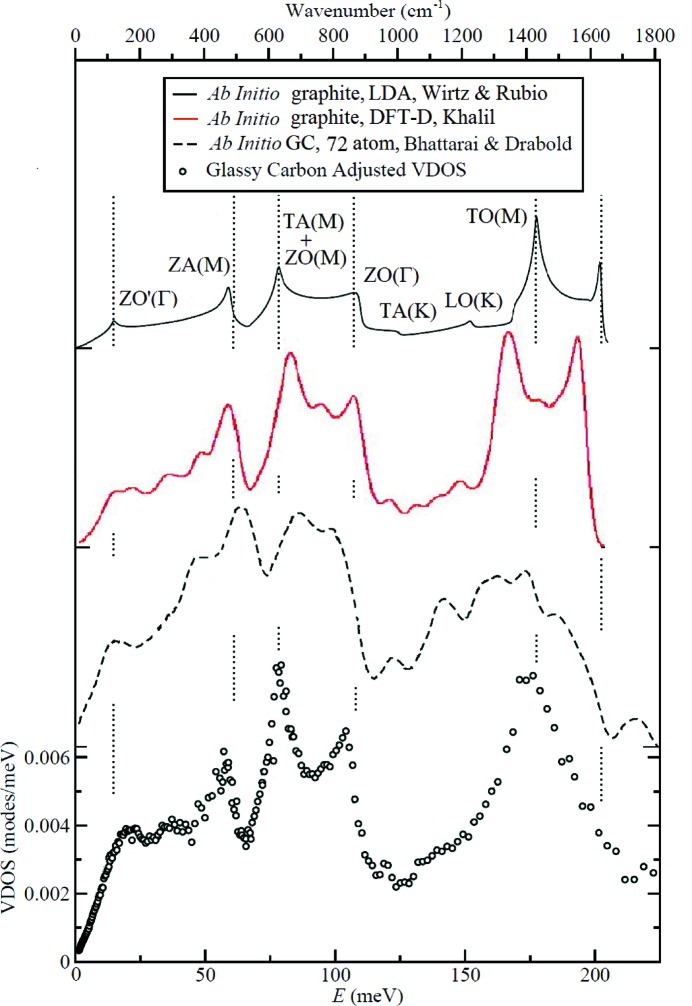
Comparison of the adjusted VDOS of low-hydrogen-content glassy carbon in this work with an *ab initio* LDA/GGA (local-density approximation/generalized gradient approximation) model for graphite (Wirtz & Rubio, 2004 ▸), an *ab initio* dispersion-corrected density functional theory (DFT-D) calculation model for graphite (Khalil, 2016 ▸) and a 72-atom *ab initio* model of GC (Bhattarai & Drabold, 2017 ▸). The mode symbol labels for graphite are adapted from Wirtz & Rubio. Their unlabeled peak near 200 meV occurs at the top of the LO band.

We can gain some insight into the measured spectrum by comparison with the theory for graphite, which is well developed (*e.g.* Wirtz & Rubio, 2004 ▸; Mounet & Marzari, 2005 ▸; Khalil, 2016 ▸). As shown in Fig. 2 ▸, beginning with lower energies, in the region below 125 meV four major spectral bands in GC around 20, 60, 80 and 100 meV match those in graphite. This gives us some assurance that the specific vibrations involved in these bands resemble those at the indicated labeled points of the graphite Brillouin zone, and the reader is referred to Wirtz & Rubio (2004 ▸) for details. The computed graphite VDOS of Kahlil below 60 meV shown in Fig. 2 ▸ appears to be in close agreement with our GC spectrum, again showing the usefulness of computational results in helping to identify vibrational features in GC. In the region above 125 meV where the resolution of the neutron spectrum is much lower (see Fig. 1 ▸), the agreement with theory for graphite is less impressive, but we may identify the broad peak around 175 meV in GC with optical modes in graphite, labeled TO(M) in Fig. 2 ▸.

In contrast to Raman and infrared spectroscopies, neutron scattering has the feature that, apart from conservation of energy and momentum, there are no other selection rules for monatomic materials. All modes, whether graphite like or arising from ‘disorder’ in the graphite or graphene lattices, will be included in the spectrum. Hence, to gain further insight into departures from graphite, probably due to disorder, we may consider Raman results. Extensive discussions of Raman spectroscopy as it relates to graphene, graphite and carbon nanotubes, including the *D* band, are given by several authors (*e.g.* Dresselhaus *et al.*, 2010 ▸; Ferrari & Robertson, 2000 ▸). For GC we turn here instead to other work (Baranov *et al.*, 1987 ▸; Wang *et al.*, 1990 ▸). In particular, Wang *et al.* studied several forms of carbon, including glassy carbon samples from Tokai Electrode Mfg Co., Nagoya, Japan (GC10, GC20 and GC30 heat-treated at 1273, 2273 and 3273 K, respectively). We may consider their Tokai GC30 sample to be close in properties to AA Type-2 material. Beginning from their lowest-energy features, using a 458 nm exciting light, they identified a *D* band (in graphite powder at 1360 cm^−1^ ≃ 168.6 meV, in GC30 at 1363 cm^−1^ ≃ 169.0 meV). There are competing interpretations of this band (see *e.g.* Baranov *et al.*, 1987 ▸; Ferrari & Robinson, 2000 ▸). Here we simply note that Wang *et al.* interpret the *D* band in terms of symmetry breaking at the edges of graphene planes in disordered materials. This band then probably adds to the TO(M)-like modes in the large band seen in Fig. 1 ▸ around 175 meV. In addition, Wang *et al.* identify an *E*_2g_ feature (in graphite powder at 1588 cm^−1^ ≃ 196.9 meV, and related modes in GC30 at 1586 cm^−1^ ≃ 196.6 meV) in the spectral region dominated by longitudinal optical modes in graphite which would contribute to the weak shoulder seen near 200 meV in GC and the stronger one in the Rols *et al.* graphite spectrum, both shown in Fig. 1 ▸.

A recent *ab initio* computational study (Bhattarai & Drabold, 2017 ▸) was made on atomic models of amorphous carbons having a variety of densities. In addition to a study of structural features, these authors have calculated the VDOS spectrum of these models. Here we choose to compare the calculated spectrum of a 72-atom model of amorphous carbon, designated c72_20ps, because it has nearly the same density as our Type-2 plate samples, namely 1.4 g cm^−3^. This is the only *ab initio* study we know of on carbon specifically having the density of GC. The result is shown in Fig. 2 ▸. In spite of the small size of this fragment and apart from a general shift to lower energies, many of the features observed in our GC measurements are reproduced in their model, and hence this approach has the potential to relate specific atomic motions in GC to spectral features. In particular, their Fig. 5 shows that the modes measured in the present study are in a region of non-localized vibrations, as indicated by small inverse-participation ratios. Their Fig. 6 shows that the contributions of *sp* ^3^ and *sp* bonding to the VDOS are much smaller than those from *sp* ^2^-bonded atoms, as is to be expected for graphitic materials. The supplemental material published with their work (available online) includes videos of atomic vibrations at several representative frequencies of the GC spectrum. Each video demonstrates a complexity arising from disorder that suggests that, while descriptions in terms of atomic motions in crystalline graphite provide useful insights, one must be cautious in applying them too literally to this glassy material.

## 4. Heat capacity measurements on a low-hydrogen-content glassy carbon sample   

### 4.1. Method and results of heat capacity measurements   

**Figure 3:**
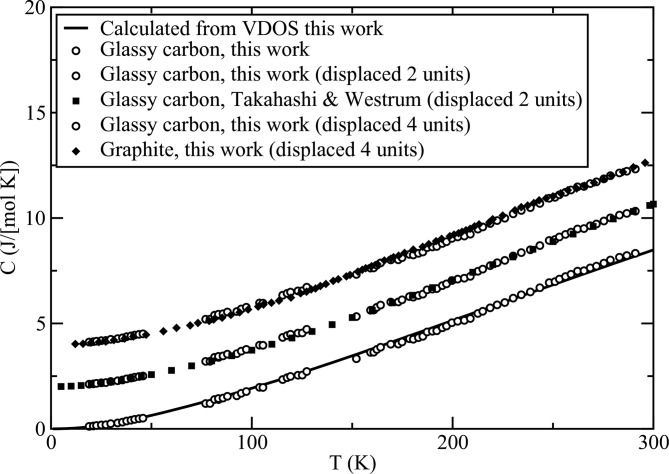
Measured specific heat capacity of the low-hydrogen-content Lannin glassy carbon sample and comparisons with theory, graphite and previous work (Takahashi & Westrum, 1970 ▸).

Fig. 3 ▸ shows heat capacity measurements made on the Lannin sample using the continuous-cooling method. Details of the method are given in the PhD dissertation of Hui Li (1993 ▸). There appears to be a smooth sigmoidal systematic discrepancy depressing the data from approximately 150 to 225 K and augmenting it from 225 to 295 K with an amplitude of about 2% at 260 K. Smaller discrepancies of this same kind were seen in measurements on other materials in this region using this technique and apparatus. The source of this discrepancy has not been definitively determined, but may be related to departure from the assumption of thermal equilibrium between the sample and addendum during the temperature ramping. Apart from this sigmoidal discrepancy, typical accumulated errors arising from thermometry, spline fitting of the tables and other causes such as thermal lag during ramping in this procedure amounted to approximately ±2% at each temperature, with a 90% confidence interval. For the Lannin sample, unreliably noisy results in the regions between 50 and 75 K and between 140 and 155 K, probably due to poor thermal contact, were discarded.

### 4.2. Discussion of heat capacity results   

In the measured temperature range, the heat capacity of glassy carbon is expected to be mainly determined by the contribution arising from vibrations of atoms within the glassy matrix with a negligible electronic contribution. Knowing the spectrum of harmonic vibrations *g*(ν), the vibrational contribution to the heat capacity is given according to standard theory by
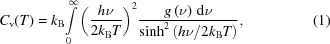
where *k*_B_ is the Boltzmann constant, *h* is Planck’s constant and *T* is temperature. The central problem in using this result is the determination of the harmonic VDOS, *g*(ν). In this work we use instead the neutron vibrational density of states which is closely related to *g*(ν) for this monatomic material. In order to use our VDOS measurements, the contributions to the spectrum that arise from multiple and multi-phonon scattering, expected to be slowly and smoothly increasing with energy, were estimated by subtracting an arbitrary background varying linearly with phonon energy *E*. The adjusted neutron VDOS shown in Fig. 2 ▸ was used to calculate the heat capacity from equation (1). While a reasonable improvement over no correction, this procedure can be expected to lead to some uncertainty in the weighting of the spectrum.

The result of the heat capacity calculation using the adjusted VDOS is shown as the solid line in Fig. 3 ▸. While agreement between this prediction and the measurement is satisfactory, the arbitrary weighting in the neutron scattering spectrum introduced to correct for multiphonon scattering precludes a more precise comparison. It is more instructive to compare the heat capacity of the Lannin sample with that of graphite (spectroscopic grade, SP-1, powder from Union Carbide Corporation), which we also measured in this work by the continuous-cooling method. Given the close similarity of the measured vibrational spectra shown in Fig. 1 ▸, the heat capacities should not differ very much, and that is found to be the case. The graphite data exhibit a *T* ^1.99 ± 0.04^ dependence in the temperature range from 20 to 60 K, as is expected by theory for quasi-two-dimensional graphite. An analysis of our glassy carbon data between 20 and 50 K gives *T* ^1.8 ± 1^, but this result is not expected to be very accurate, given the paucity of data points in this region. Our heat capacity measurement is also compared with measurements made on a GC30 rod sample from Tokai, showing substantial agreement, considering the sample and methodological differences (Takahashi & Westrum, 1970 ▸). Those workers report *T* ^2.04^ for the Tokai glassy carbon sample in the more useful low-temperature range between 5 and 30 K, in substantial agreement with the result for graphite.

## 5. Summary   

Commercial Alfa Aesar Type-2 glassy carbon plates have been developed as absolute intensity calibration standards in SAXS measurements and designated as new NIST Standard Reference Material SRM 3600. We have further characterized this material by sampling a plate provided to us from APS and found that it has a hydrogen content of (4.8 ± 0.2) × 10^−4^ (mol H)/(mol C). We have measured the vibrational spectrum of a collection of these plates by neutron inelastic scattering. The measured spectrum strongly resembles that of graphite, allowing us to identify the likely atomic motions of major spectral features in this glassy material. We have measured the heat capacity of a low-hydrogen-content glassy carbon sample from 20 to 295 K and of graphite powder in the same range for comparison, finding substantial agreement. This agreement is to be expected from the vibrational spectral similarity of these two materials. Our measured vibrational spectrum of this low-hydrogen-content glassy carbon is used to calculate its heat capacity, and a favourable comparison is made between theory and experiment.

## Footnotes

1 Certain commercial entities, equipment or materials may be identified in this document in order to describe an experimental procedure or concept adequately. Such identification is not intended to imply recommendation or endorsement by the National Institute of Standards and Technology, nor is it intended to imply that the entities, materials or equipment are necessarily the best available for the purpose.

## References

[bb1] Albers, P. W., Weber, W., Möbus, K., Wieland, S. D. & Parker, S. F. (2016). *Carbon*, **109**, 239–245.

[bb2] Allen, A. J., Zhang, F., Kline, R. J., Guthrie, W. F. & Ilavsky, J. (2017). *J. Appl. Cryst.* **50**, 462–474. 10.1107/S1600576717001972 PMC5377342 28381972

[bb3] Baranov, A. V., Bekhterev, A. N., Bobovich, Ya. S. & Petrov, V. I. (1987). *Opt. Spectrosc. (USSR)*, **62**, 612–616.

[bb4] Bhattarai, B. & Drabold, D. A. (2017). *Carbon*, **115**, 532–538.

[bb5] Dresselhaus, M. S., Jorio, A. & Saito, R. (2010). *Annu. Rev. Condens. Matter Phys.* **1**, 89–108.

[bb6] Ferrari, A. C. & Robertson, J. (2000). *Phys. Rev. B*, **61**, 14095–14107.

[bb7] Franklin, R. E. (1951). *Proc. R. Soc. London Ser. A*, **209**, 196–218.

[bb8] HTW (2017). *Glassy Carbon SIGRADUR*, http://www.htw-germany.com/technology.php5?lang=en&nav0=2&nav1=16.

[bb9] Jurkiewicz, K., Duber, S., Fischer, H. E. & Burian, A. (2017). *J. Appl. Cryst.* **50**, 36–48.

[bb10] Kamitakahara, W. A. (1996). *J. Phys. Chem. Solids*, **57**, 671–676.

[bb11] Kamitakahara, W. A., Lannin, J. S., Cappelletti, R. L., Copley, J. R. D. & Li, F. (1992). *Physica B*, **180–181**, 709–710.

[bb12] Khalil, R. M. A. (2016). *Chin. J. Phys.* **54**, 890–894.

[bb14] Li, F. & Lannin, J. S. (1992). *Appl. Phys. Lett.* **61**, 2116–2118.

[bb13] Li, H. (1993). PhD dissertation, Ohio University, Athens, OH, USA, https://inis.iaea.org/search/search.aspx?orig_q=RN:27025293.

[bb15] Mildner, D. F. R. (1974). PhD dissertation, University of Michigan, Ann Arbor, USA, http://deepblue.lib.umich.edu/bitstream/2027.42/6614/5/bac9480.0001.001.pdf.

[bb16] Mildner, D. F. R. & Carpenter, J. R. (1982). *J. Non-Cryst. Solids*, **47**, 391–402.

[bb17] Mounet, N. & Marzari, N. (2005). *Phys. Rev. B*, **71**, 205214.

[bb18] Parker, S. F., Imberti, S., Callear, S. K. & Albers, P. W. (2013). *Chem. Phys.* **427**, 44–48.

[bb19] Paul, R. L., Şahin, D., Cook, J. C., Brocker, C., Lindstrom, R. M. & O’Kelly, D. J. (2015). *J. Radioanal. Nucl. Chem.* **304**, 189–193.

[bb20] Rols, S., Benes, Z., Anglaret, E., Sauvajol, J. L., Papanek, P., Fischer, J. E., Coddens, G., Schober, H. & Dianoux, A. J. (2000). *Phys. Rev. Lett.* **85**, 5222–5225. 10.1103/PhysRevLett.85.5222 11102226

[bb21] SRM 3600 (2017). National Institute of Standards and Technology, Certificate of Analysis SRM 3600, https://www-s.nist.gov/srmors/view_detail.cfm?srm=3600.

[bb22] Takahashi, Y. & Westrum, E. F. Jr (1970). *J. Chem. Thermodyn.* **2**, 47–54.

[bb23] Udovic, T. J., Brown, C. M., Leão, J. B., Brand, P. C., Jiggetts, R. D., Zeitoun, R., Pierce, T. A., Peral, I., Copley, J. R. D., Huang, Q., Neumann, D. A. & Fields, R. J. (2008). *Nucl. Instrum. Methods Phys. Res. A*, **588**, 406–413.

[bb24] Wang, Y., Alsmeyer, D. C. & McCreery, R. L. (1990). *Chem. Mater.* **2**, 557–563.

[bb25] Wirtz, L. & Rubio, A. (2004). *Solid State Commun.* **131**, 141–152.

[bb26] Zhang, F., Ilavsky, J., Long, G. G., Quintana, J. P. G., Allen, A. J. & Jemian, P. R. (2010). *Metall. Mater. Trans. A*, **41**, 1151–1158.

